# Poster Session II A329 SLEEP QUALITY AS MEDIATING FACTOR BETWEEN ADVERSE CHILDHOOD EXPERIENCES AND IBS SYMPTOM SEVERITY: AN EXPLORATORY STUDY

**DOI:** 10.1093/jcag/gwaf042.329

**Published:** 2026-02-13

**Authors:** S Busse, N Haskey, Y Nasser, M Raman

**Affiliations:** Biology, The University of British Columbia, Vancouver, BC, Canada; Biology, The University of British Columbia, Vancouver, BC, Canada; Medicine, University of Calgary Cumming School of Medicine, Calgary, AB, Canada; Medicine, University of Calgary Cumming School of Medicine, Calgary, AB, Canada

## Abstract

**Background:**

Approximately 18% of Canadians live with Irritable bowel syndrome (IBS). Few treatments concurrently address modifiable risk factors in addition to symptoms. Adverse Childhood Experiences (ACEs) have been shown to worsen IBS severity through shared neurointestinal pathways, however, mediating mechanisms and therapeutic targets have not been explored, limiting its clinical use.

**Aims:**

To determine the types of adverse childhood experiences associated with IBS symptom severity and identify potential mediating factors.

**Methods:**

We report preliminary results of a prospective exploratory survey that included adults ≥ 18 years of age diagnosed with IBS using conventional criteria recruited through in-person and online advertisements after July 2025. Participants completed a self-administered REDCap survey including demographic information and seven validated questionnaires, IBS Symptom Severity Scale (IBS-SSS), Adverse Childhood Experience Questionnaire (ACE-Q), Early Trauma Self-Report Short Form (ETI-SF-SR), Brief Pittsburgh Sleep Quality Index (B-PSQI), Patient Health Questionnaire-8 (PHQ-8), Perceived Stress Scale-10 (PSS-10) and Generalized Anxiety Disorder-7 (GAD-7).

**Results:**

Nearly half of participants (45%) reported exposure to at least four ACEs, with 41% specifically reporting more than 4 emotional ACEs (ETI-SF-SR sub score) (Table 1). Contrary to expectation, ACE-Q (r = 0.111, p = 0.567), total ETI-SF-SR (r = 0.052, p = 0.788) and ETI-SF-SR sub types, B-PSQI (r = 0.226, p = 0.239), PHQ-8 (r = 0.143, 0.459), PSS-10 (r = 0.117, p = 0.554 and GAD-7 (r = 0.092, r = 0.628) showed no significant correlation with IBS-SSS. However, the emotional trauma sub score of ETI-SF-SR (r = 0.414, p = 0.026), total ETI-SF-SR score (r = 0.376, p = 0.044) and ACE-Q score (r = 0.392, p = 0.035) showed statistically significant association with B-PSQI (with higher B-PSQI score indicating poorer sleep quality).

**Conclusions:**

While ACEs and IBS symptom severity were not associated in this pilot study, ACEs negatively influenced sleep quality. Future investigation should explore sleep quality as a potential mediating factor between ACEs and IBS symptoms.

**Funding Agencies:**

None

**A329 Table 1:** Total ACE, Emotional Sub score and Sleep Quality of Participants

Outcome	N = 29^1^
*Total ACEs (ACE-Q)*	
0	9 (31%)
1 - 3	7 (24%)
4+	13 (45%)
*Emotional Trauma ACEs (ETI-SF-SR Emotional Trauma Sub Score)*	
0	8 (28%)
1 - 3	9 (31%)
4 - 5	12 (41%)
*Sleep Quality (B-PSQI)*	
Good Quality (B-PSQI ≤ 5)	8 (28%)
Poor Quality (B-PSQI > 5)	21 (72%)

1 n (%)

